# Supplementary figures and images for: Smart Farming Enhances Bioactive Compounds Content of Panax ginseng on Moderating Scopolamine-Induced Memory Deficits and Neuroinflammation
Source: Plants (Basel). 2023 Feb 1;12(3):640. doi: 10.3390/plants12030640 (PMC9920294; doi:10.3390/plants12030640)

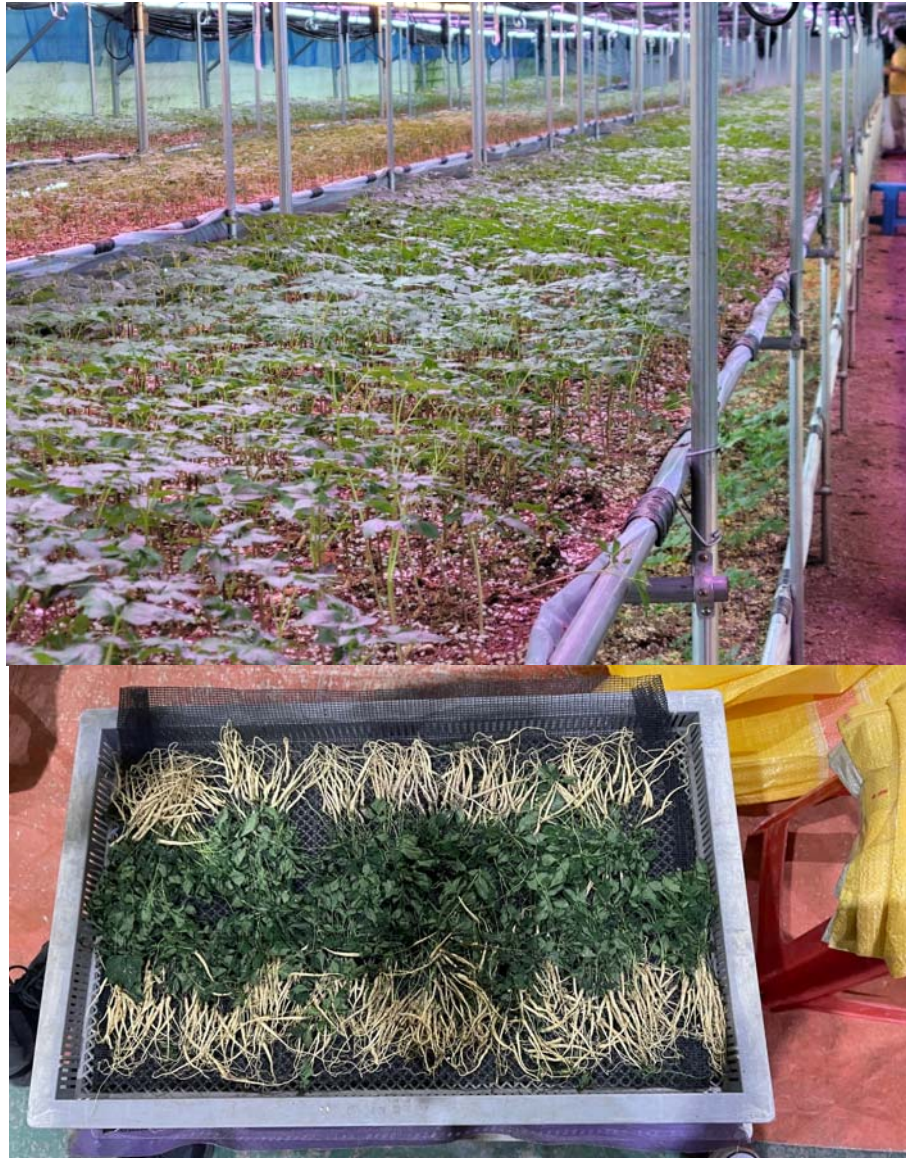

Figure S1. smart farming system and *P.ginseng* sprouts.

Supplement: Supplementary file 1 [file plants-12-00640-s001.zip › plants-2157156-supplementary.pdf]
